# Vortex Formation with a Snapping Shrimp Claw

**DOI:** 10.1371/journal.pone.0077120

**Published:** 2013-11-14

**Authors:** David Hess, Christoph Brücker, Franziska Hegner, Alexander Balmert, Horst Bleckmann

**Affiliations:** 1 Institute of Mechanics and Fluid Dynamics, Technical University of Freiberg, Freiberg, Saxony, Germany; 2 Institute of Zoology, University of Bonn, Bonn, North Rhine-Westphalia, Germany; University of Manchester, United Kingdom

## Abstract

Snapping shrimp use one oversized claw to generate a cavitating high speed water jet for hunting, defence and communication. This work is an experimental investigation about the jet generation. Snapping shrimp (*Alpheus-bellulus*) were investigated by using an enlarged transparent model reproducing the closure of the snapper claw. Flow inside the model was studied using both High-Speed Particle Image Velocimetry (HS-PIV) and flow visualization. During claw closure a channel-like cavity was formed between the plunger and the socket featuring a nozzle-type contour at the orifice. Closing the mechanism led to the formation of a leading vortex ring with a dimensionless formation number of approximate ΔT*≈4. This indicates that the claw might work at maximum efficiency, i.e. maximum vortex strength was achieved by a minimum of fluid volume ejected. The subsequent vortex cavitation with the formation of an axial reentrant jet is a reasonable explanation for the large penetration depth of the water jet. That snapping shrimp can reach with their claw-induced flow. Within such a cavitation process, an axial reentrant jet is generated in the hollow cylindrical core of the cavitated vortex that pushes the front further downstream and whose length can exceed the initial jet penetration depth by several times.

## Introduction

**Figure 1:**
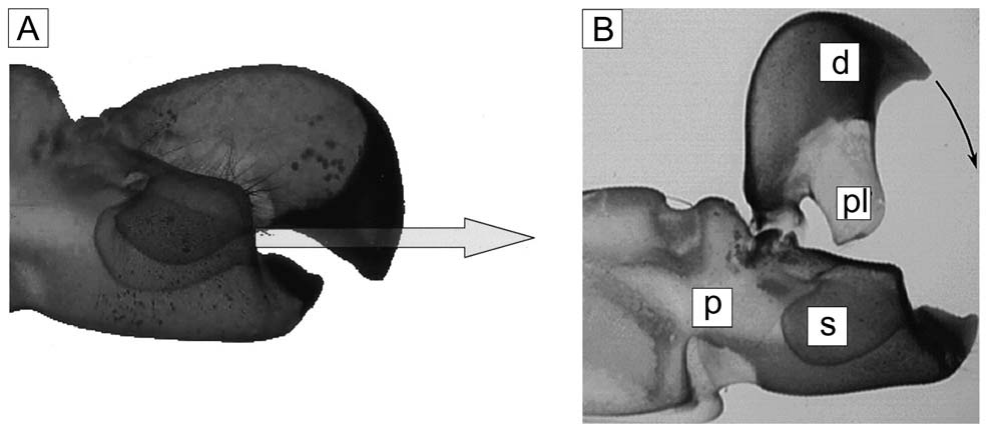
Pictures taken from Versluis et al. [1]. The left picture (A) shows a photograph of the snapper claw, made transparent by a special chemical process in nearly closed position. The arrow indicates the direction of the water jet exiting the flow channel built between the plunger and the socket in the late stage of closure. Note the difference of the angle between the flow channel near the orifice and the angle of the arrow indicating the jet direction. The picture B (by B. Seibel) describes the geometry and names the parts as followed. d: dactyl pl: plunger p propus: s: socket.

Snapping shrimp of the family *Alpheidae (Decapoda, Caridea)* form a water jet by rapid closure of their snapper claw [1]. One side of the claw is the dactyl while the other side is the propus [2]. The dactyl possesses a stopper-like tooth (the plunger) which fits into a socket of the propus. Volz [1] described that a rapid jet of water is formed when the dactyl plunger is driven into the propus socket, displacing water which escapes through a narrow anterior groove (see also figure 1 A and B). Using high-speed imaging, Herberholz & Schmitz [4] showed that water jet distance, width and velocity are correlated to snapper claw size. In their experiments a drop of ink was positioned in front of the open (completely cocked) snapper claw immediately before claw closure, and was filmed via high-speed imaging as the drop was displaced by the resulting water jet. Distance and width of the jet showed a significant correlation and both were larger in animals with larger snapper claws. Typical values of jet penetration distance were in the order of the claw length (>10 mm). Results also showed that the initial high water jet velocity already decreases after the first millisecond and drops to 0.2 m/s within the first 20 ms. The average value of maximum water jet velocity was 6.5 m/s. This velocity matched the mean claw closure velocity of 5.9 m/s. Originally, it was believed that the sound occurring during closure of the claws is generated by the two surfaces hitting each other. However, the discovery of the cavitating flow showed that the sound is in fact caused by the violent collapse of a large cavitation bubble. This bubble is generated by the tensile forces of the water jet that forms when the shrimp's snapper claw snaps shut [3]. This was shown by using ultrahigh-speed imaging of the snapper claw closure at 40500 frames per second. In figure 1 a picture of this claw is shown and most important parts are named (still image). A temporal analysis of the sound recordings and the high-speed images showed that no sound was associated with claw closure. The pressure profile over time was estimated by theoretical arguments to justify the conclusion by Versluis et al. [3]. They estimated the velocity of the water jet from the speed of the cavitation bubble. This velocity implied a pressure drop from ambient pressure of about 10^5^ Pa. However, there is limited information on the actual temporal and spatial shape of the velocity field and consequently, on the pressure field. The aim of the present paper was to fill this gap and to analyse in detail the jet flow generated by the closing of the snapper.

## Materials and Methods

### Claw morphology

**Figure 2:**
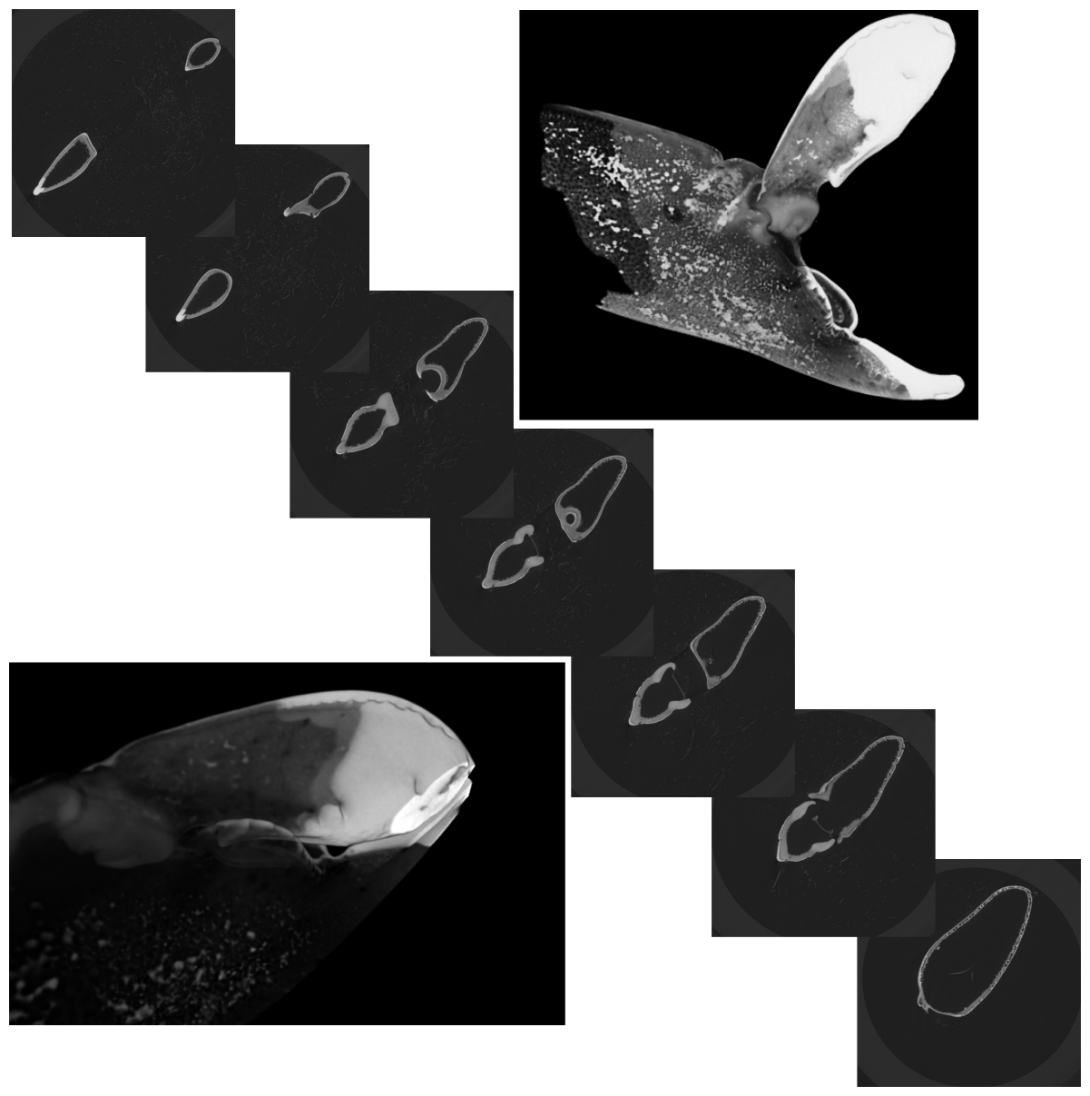
The images show a slice-wise arrangement of the μCT images from the opened claw of *Alpheus bellulus*. The top left slice represents the opened end of the claw, the bottom down image displays the last extracted slice at the joint of the propus and the dactyl (bottom right image) The images in the top right and down left corner show 3D reconstructions of an opened and closed claw.

Within this work our major focus was to understand of the main ideas of the jet formation and its propagation in a snapping shrimp and not a comparison within different specimen. Furthermore, the effort needed for larger number of specimen would be extensive, since the following procedure to generate the transparent model geometry needed to be done for each individual. Therefore, we decided to focus on the morphology of a typical specimen of snapping shrimp *A. bellulus*. The research with the animals reported herein was performed under the guidelines established by the German animal protection law. The shrimps were purchased form a commercial dealer (Mrutzek Meereaquaristik GmbH, Ritterhude, Germany) and kept solitary in saltwater aquaria. The shrimps were allowed to moult at least twice to ensure that the snapper claws were fully differentiated. Snapper claws were obtained by gently pinching und pulling the snapper claw in the merus region to induce autotomy of the claw. Parts of the proximal propus were removed with a scissor and a pincher to reduce specimen size for the micro X-ray computed tomography (μCT) in order to increase spatial resolution. The snapper claws of *Alpheus bellulus* were cleaned with distilled water to remove salt residues and then critical-point-dried (CP 020, Balzers Union GmbH, Aβlar, Germany). The dried snapper claws were placed into small plastic vials and stabilized with cotton wool. The plastic vials were observed twice in the μCT (μCT 20, Scanco Medical AG, Bassersdorf, Germany). Firstly, claws were scanned when completely closed. Afterwards, the claws were rehydrated in a 70%-ethanol/water solution and the dactyl was raised to the maximum open position. Claws were dried again and scanned for a second time. Example images of the μCT scans of a typical claw are shown in figure 2. A histogram cut and a median filter were applied to each slice to remove flaws in the resin and background noise.

The diagonal arranged images in figure 2 show a slice-wise arrangement of the μCT images from the claw in opened situation. The top left slice represents the distal end of the claw while the bottom right one is at the joint of the propus and the dactyl. When following the slices one can see that the dactyl and the propus get bigger and form the details of the plunger and the socket. Finally, the stack of slices was imported into Amira® which allowed visualising and rendering the claw in 3D. The 3D images of the claw in opened and closed situation can be seen in the corners of figure 2.

### Reconstruction of flow chamber being formed when dactyl plunger moved into propus socket

**Figure 3:**
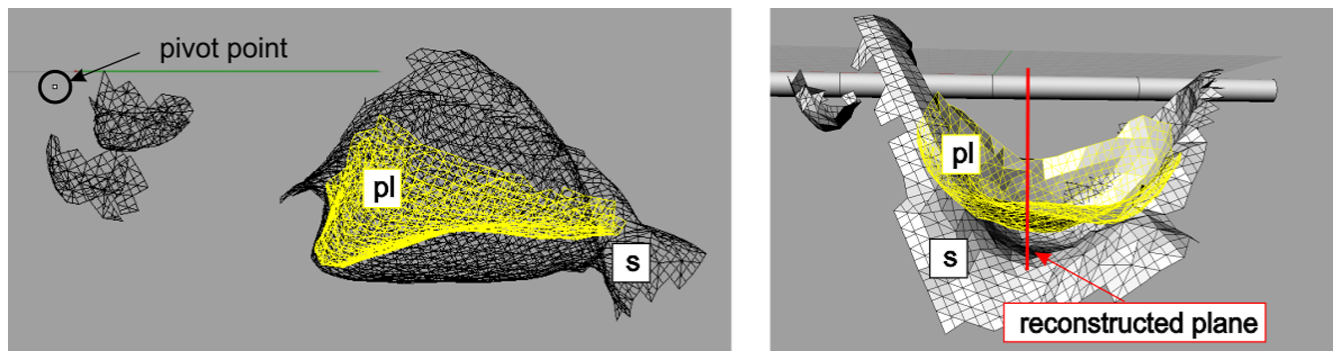
Cut out of the functional geometries forming the flow channel in side view (left) and front view (right). The rotation axis is highlighted. PL (stopper-like tooth of the dactyl plunger in yellow, socket of propus in black). Rotational motion is in the paper-plane on the left-hand side (x-y-plane). The location of the centre plane along the long axis of the flow channel is indicated by the yellow line in the front view.

The 3D surface data of the claw were then transferred via the interface of a virtual reality modelling language (VRML) file into a computer-aided design software (CAD software Unigraphics NX8). Here, the main functional geometries of the claw were separated into the surface contours of the plunger, the socket and their connections to the pivot axis. Finally, the CAD program allowed us to rotate the surfaces relative to each other around the pivoting axis as in nature, when the claw performed the closing motion. The maximum opening angle (α) of the claw was measured from the CAD data to Δα = 81.5°. When the plunger was rotated in the CAD model into the socket a cavity was formed between the walls of both with a remaining outlet at the front end of the claw. From the CAD model we could see, that the last phase of the snapping process, where the opening angle Δα is less than 20°, is most important for jet formation. The plunger starts working as a displacement piston and the closing gaps between the plunger and the socket on the lateral surfaces behave as a sealing. The cavity geometry for the closed situation, when the plunger tooth finally hits the contour of the propus socket is shown in figure 3 with the plunger contour highlighted in yellow.

**Figure 4:**
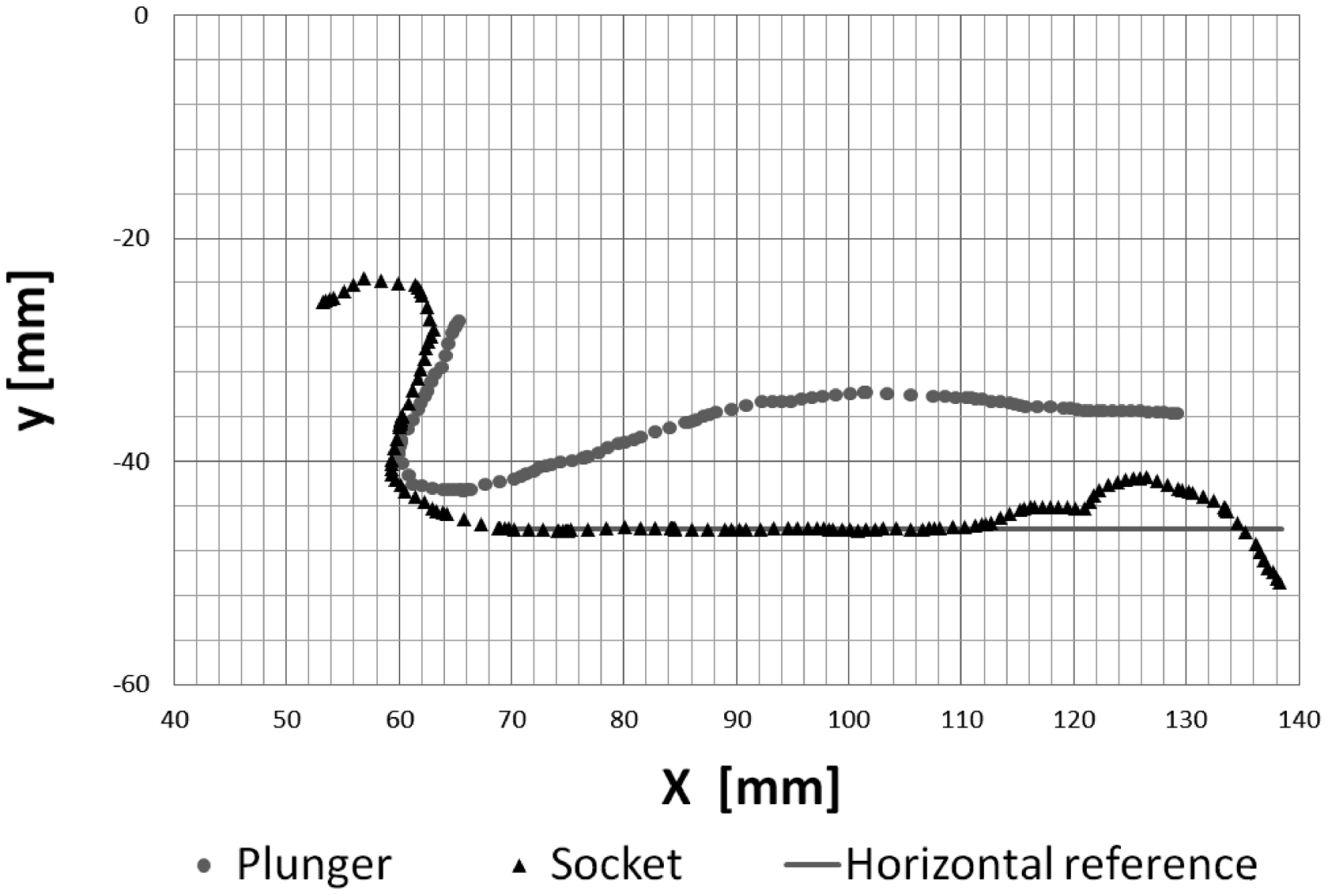
Contour points in the cross-sectional plane (x-y-plane) along the long axis of the flow channel in the closed state of the claw. The flow channel is formed by the gap between the contours of the dactyl plunger (grey dots) and the socket (black triangles) in the propus. The closed position is reached when the tooth of the plunger is in contact with the socket on the left-hand side. Note that the pivot point would be x|y = 0|0.

A two dimensional slice along the centre plane of the cavity and the orifice is shown for clarification of the contour of the flow channel. This plane is approximately a left-right-symmetry plane of the cavity along the long axis of the flow channel, see figure 4. On the right-hand side, a nozzle contour has formed which represents the orifice of the channel where the fluid within the chamber is ejected into the ambient water. Note that the long axis of the cavity built between both walls (plunger and socket) is 4–5 times larger than the lateral extension of the cavity. In addition, the curvature radius of the walls in the cross-sectional plane perpendicular to the long axis is large in comparison to the cavity height. These observations gave reason to simplify the cavity geometry in first approximation as a flow chamber with planar side-walls and with rectangular cross-sections at all positions along the flow path to the orifice. The procedure how the model claw was built from these data is described in the following.

### The transparent claw model

**Figure 5:**
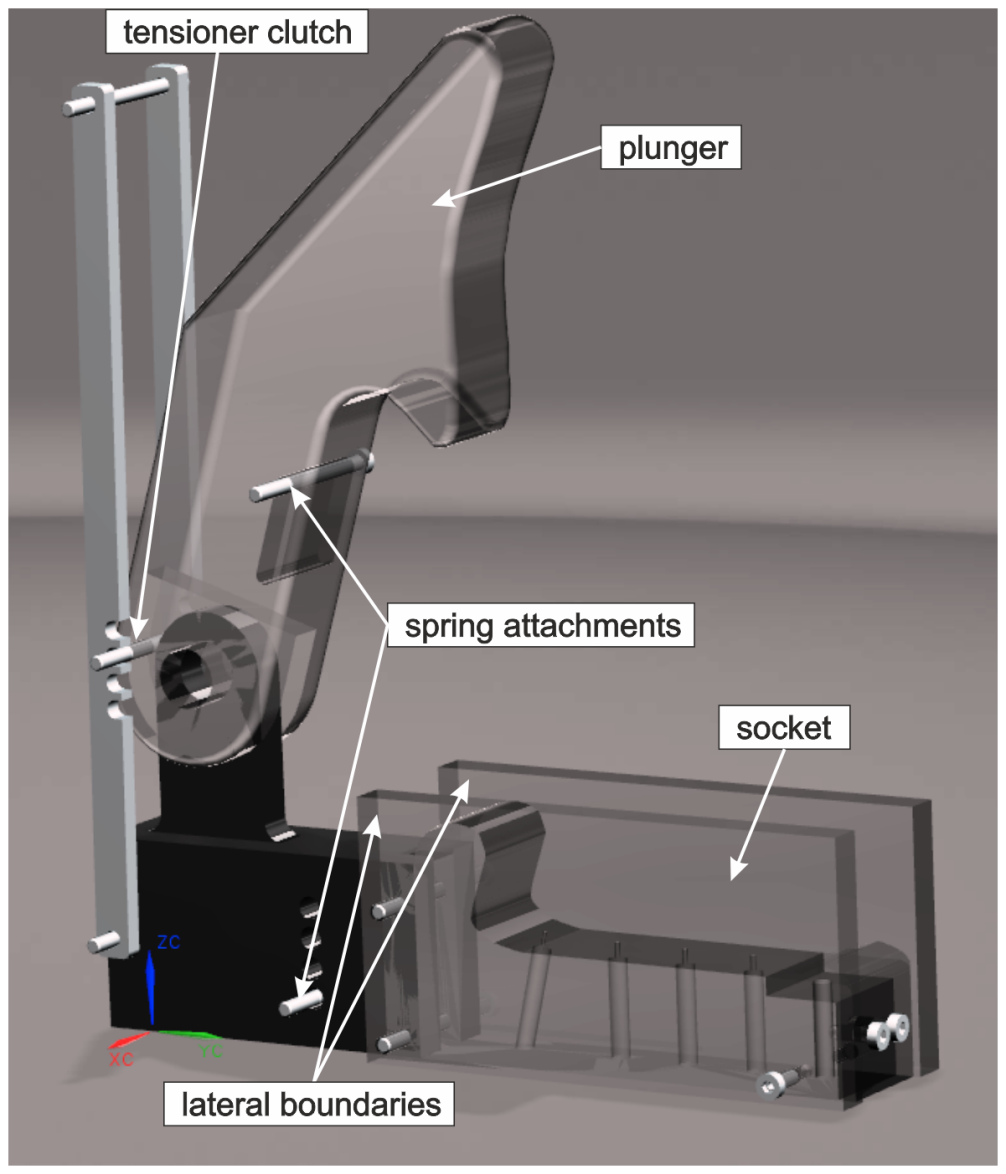
CAD-model of the enlarged claw model with the movable plunger in maximum vertical opening position and the socket fixed at the floor with two transparent side-walls made from Perspex which allowed optical access into the cavity. At the pivot point an axis is included to the two bodies, so the plunger can rotate to the closed position. To accelerate the plunger to the required angular velocity a spring is stretched between the stopper and the plunger.

Flow measurements of the jet formation in the cavity between dactyl plunger and propus socket require the manufacture of a transparent model, which allows optical access from at least two sides. In addition, we took advantage of using an enlarged model (scale: 70∶1) which makes flow visualization of jet evolution in the closing phase much more practicable since the time-scale of the process in the enlarged geometrical scale is stretched to a much larger closure period (see below). As in nature, a plunger was build which rotated around a fixed pivoting axis and moved into a socket. From our own measurements and motion images shown in Versluis et al. [3] it can be concluded that the dactyl motion is by far the most dominant part in the closure process. Therefore any motion of the propus socket can be neglected in first approximation and the model can be fixed for practical reasons. A more detailed discussion of the consequences is provided in the discussion section. The plunger was clamped in the maximum opening position and a spring mechanism was released at a specific moment which started the motion of the plunger (see figure 5) into the socket.

As discussed in the previous section the contours along the long axis of the cavity were taken as the reference contours in the x-y-plane representing the plunger wall (upper contour) and socket wall (lower contour) of the model, see figure 4. Since, these points were extracted from the symmetry plane of the claw, a quasi 2D Flow in this region is expected. For the same reason and as argued above we simplified the model claw as a plunger and socket with a quasi 2D geometry with planar sidewalls. Therefore we obtained parallel walls of plunger and socket in the z-direction which then form a rectangular cross-section of the flow channel in the x-z-plane. The measurement points shown in figure 4 were used as supporting points for curve fits of the model plunger and socket wall contours. Therefore, the grey points of the plunger and the black triangles representing the socket contour in the x-y centre plane were fitted with approximating splines (as for example the grey line for the horizontal reference). The width of the flow channel was adjusted to the mean lateral extension of the cavity and was *W* = 3 cm in the model. The total length of the flow channel, measured from the tooth of the plunger sitting on the socket downstream to the minimum cross-section in the nozzle, was *L* = 10 cm. These data were then used for construction of the model pieces using the CAD program. The side walls of the socket were built in form of parallel transparent plates to avoid refractive index problems and optical distortions. A gap of 3 mm between the inner socket side walls and the outer plunger side walls was left on each side of the channel so that the plunger motion was mainly controlled by the fluid pressure building up in the channel during jet formation and not influenced by any squeeze flow effects in the gap of the side walls. An image from the CAD model is shown in figure 5.

### The fluid-dynamical parameters

The resulting flow parameters in the model were based on the theory of mechanical and dynamical similarity of cavity flow in nature vs. the model. Therefore, the dimensionless numbers *Re* and *Sr* need to be constant. The Reynolds number *Re* defines the ratio of inertial forces to viscous forces in the fluid [5] and is determined as follows:
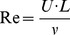
(1)Wherein is ν the kinematic viscosity of the fluid, *L* the characteristic dimension of the flow channel (length *L* of the cavity in closed situation) and *U* the characteristic velocity (typical jet velocity). With typical values of *U*≈17 m/s, *L*≈1.4 mm and ν≈1 mm^2^/s, the typical Reynolds number of the flow is in the of order of 24.000. The Strouhal number *Sr* represents a dimensionless time-scale of the closure process and is defined with *T* as the closure time of the shrimp claw using the following equation [5]

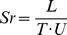
(2)The resulting Strouhal number with a typical closure time of *T*≈500 µs is 0.17. To match the refractive index of the transparent material of the model with the fluid a water/glycerine mixture was chosen. A mixing ratio of 56∶44% of glycerine was used. The kinematic viscosity ν of the fluid at room temperature was 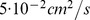. An additional ratio is defined by the Euler-number, which relates the pressure Δ*p* to the characteristic dynamic pressure 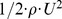:
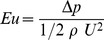
(3)Combining the equations of constant parameters in the model and the original (with subscript “O”) the following ratios can be found:
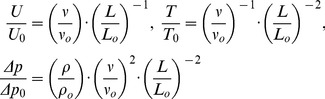
(4)The resulting parameters of the flow in the scaled-up model are summarized in Table 1

**Table 1:** Parameters of the scaled-up (70∶1) model case.

	*Snapping shrimp*	*Model claw*	*Scaling ratio*
Geometry	*L_O_*≅1.41 mm	*L*≅100 mm	*L/L_O_*≅70
Medium-	ν_O_≅1 cm^2^ s^−1^	ν≅5 cm^2^ s^−1^	*ν/ν_O_*≅5
properties	ρ_O_≅1 g cm^−3^	ρ≅1 g cm^−3^	*ρ/ρ_O_*≅1
Closure time	*T_O_*≅500 µs	*T*≅0.5 s	*T/T_O_*≅1000
Flow velocity	*U*_O_≅17 m s^−1^	*U*≅1 m s^−1^	*U/U_O_*≅1/17
Dyn. pressure	Δ*p*_O_≅10^5^ Pa	Δ*p*≅500 Pa	Δ*p*/Δ*p*_O_≅1/200
Reynolds Nr.	≅24000	≅24000	≅24000
Strouhal Nr.	≅0,17	≅0,17	≅0,17

Furthermore, in principle also the cavitation number (Ca) needs to be kept constant, when cavitation effects may play a role in the flow process. The cavitation number is defined as followed:
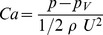
(5)where p is the absolute pressure and p_V_ represents the liquid's vapour pressure. The cavitation number characterises the potential whether a high-speed flow would reach such low pressures that the fluid cavitates or not.

According to fluid mechanical similarity of flow and forces these dimensionless numbers need to be kept constant in original claw and model claw flow. As a first step, this work aims to understand how the flow is generated within the closing claw of the snapping shrimp. Cavitation effects are not involved in this initial formation process, therefore only the Reynolds- and Strouhal-number are relevant for this flow situation. With the given liquid properties in the model experiments the characteristic flow velocity is largely reduced and the closing time is largely increased, see Table 1. In order to keep also the cavitation number constant, it would be necessary to change the vapour pressure of the liquid (in our case a water-glycerine mixture), which is not possible. Hence, the enlarged model is able to show the main flow characteristics, but it would not show any cavitation effects. Therefore, additional experiments were carried out with a non-transparent down-scaled version of the model claw (in scale of 2∶1 to the original). Here the generated pressures were low enough to generate cavitation in water. Herein, flow visualisation was used to show the temporal evolution of the flow outside of the claw where the jet entered into the ambient fluid.

### Measurement set up and procedure

**Figure 6:**
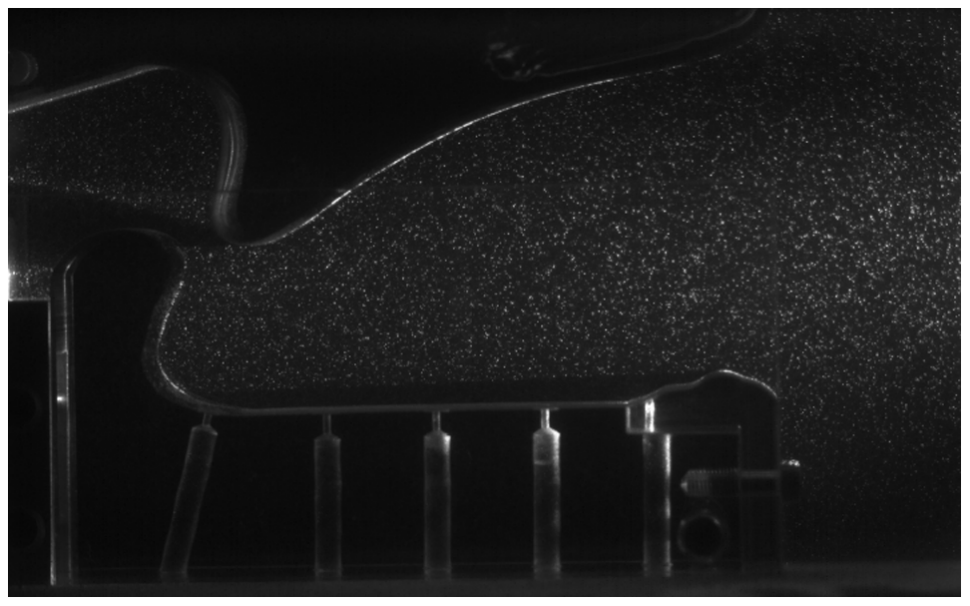
Typical PIV-image taken with the high-speed camera in the closure phase of the plunger.

The 70∶1 enlarged model of the claw was placed in a transparent glass tank (width 200 cm, height 50 cm and depth 50 cm) filled with the water-glycerine mixture. The model was completely submerged and fixed at the floor of the tank. A high-speed Laser (Pegasus, New Wave, Nd∶YAG laser, 10 mJ per pulse at 1 kHz repetition rate) was placed next to the tank with a light arm, which illuminated a 2 mm thin sheet in the fluid which was oriented in the x-y-plane in the centre of the model. Small tracer particles (Vestosint, BASF, mean diameter 50 µm) were added to the fluid. The light scattered from the particles was recorded with a high-speed Camera (Phantom V12.1, Vision Research) at a viewing angle in direction normal to the light-sheet plane, representing a side-view into the cavity. Frame rates were adjusted to 1000–5000 fps, depending on the speed of closure controlled by the spring constant. A typical picture taken with the camera at the moment of entrance of the plunger into the sidewalls of the socket is shown in figure 6 and a visualisation of the experiment can be seen in Video S1.

The motion of the plunger was determined by image processing of a reference marker placed on the surface of the plunger (light-emitting diode, LED bulb) and calculating the angular position over time relative to the rotatory motion of the plunger. The reference position was defined as the position where the plunger was in a closed position corresponding to an angle of Δα = 0. The maximum opening position was at an angle of Δα = 81.5°.

The measurements of the flow and the kinematics of the plunger during closure of the model claw were repeated at least three times showing reproducible results. The closing time was in the range *T* = 500±5 ms which shows a variation of order of 1% of the total period. Since the temporal profile of the plunger motion determines the flow evolution, flow differences between different realisations stem mainly from the small variations of the motion profiles. Hence, all the results shown in this work are discussed on one representative experiment.

## Results and Discussion

**Figure 7:**
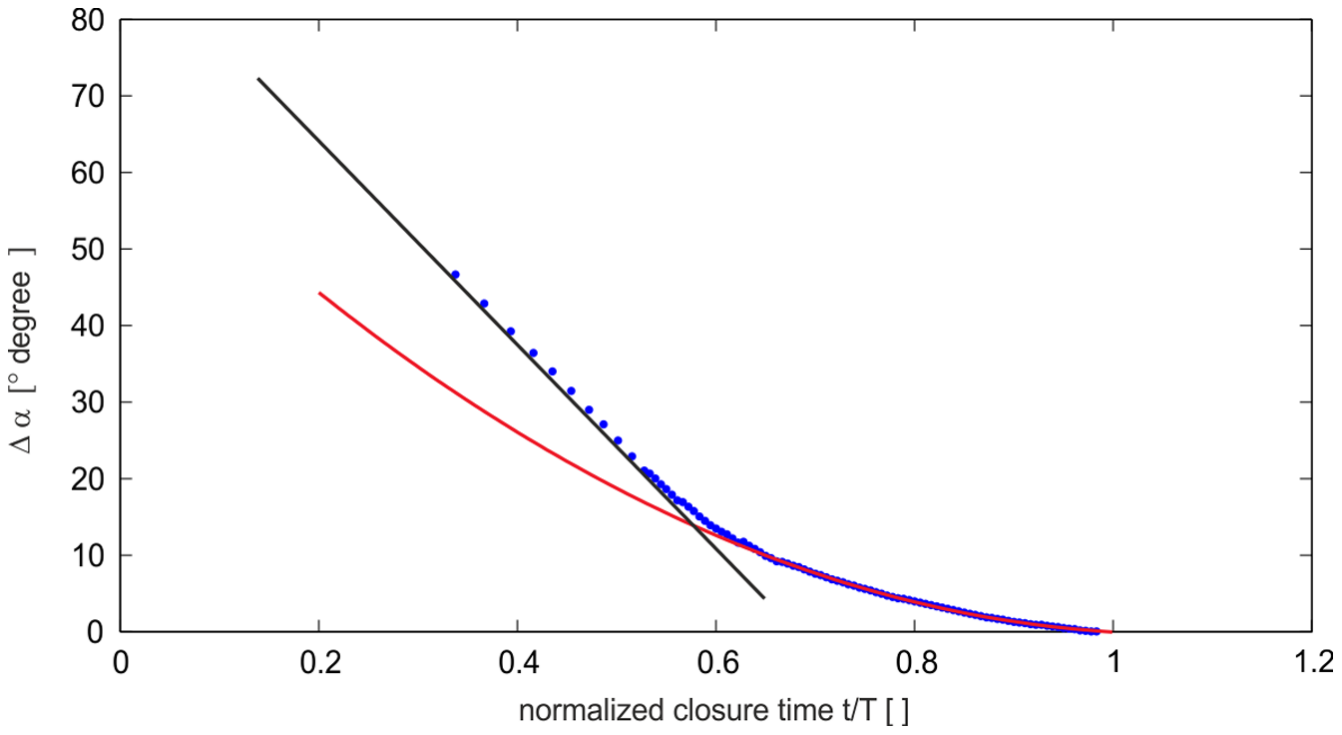
Temporal evolution of the angle of the relative angle between plunger and stopper in the closure cycle. The red curve represents a 2nd order polynomial fit to the measurements in the late stage of closure. The black line represents the linear trend of the early phase of closure.

The resulting profile for the claw closure process is shown in figure 7. The motion is represented by two distinct phases. In the first phase after release of the plunger fixation mechanism, the plunger accelerates (not shown here) and approaches the first phase of constant angular velocity t*>0.3 with t* representing the time *t* normalized with the period *T* when full closure of the claw is reached. At t*≈0.7 this phase changes into a deceleration phase which follows a parabolic shape (red curve) with a constant angular deceleration. Full closure of the claw is then reached at t* = 1. Note, that a careful inspection of the motion after closure reveals a small but distinct rebound.

**Figure 8:**
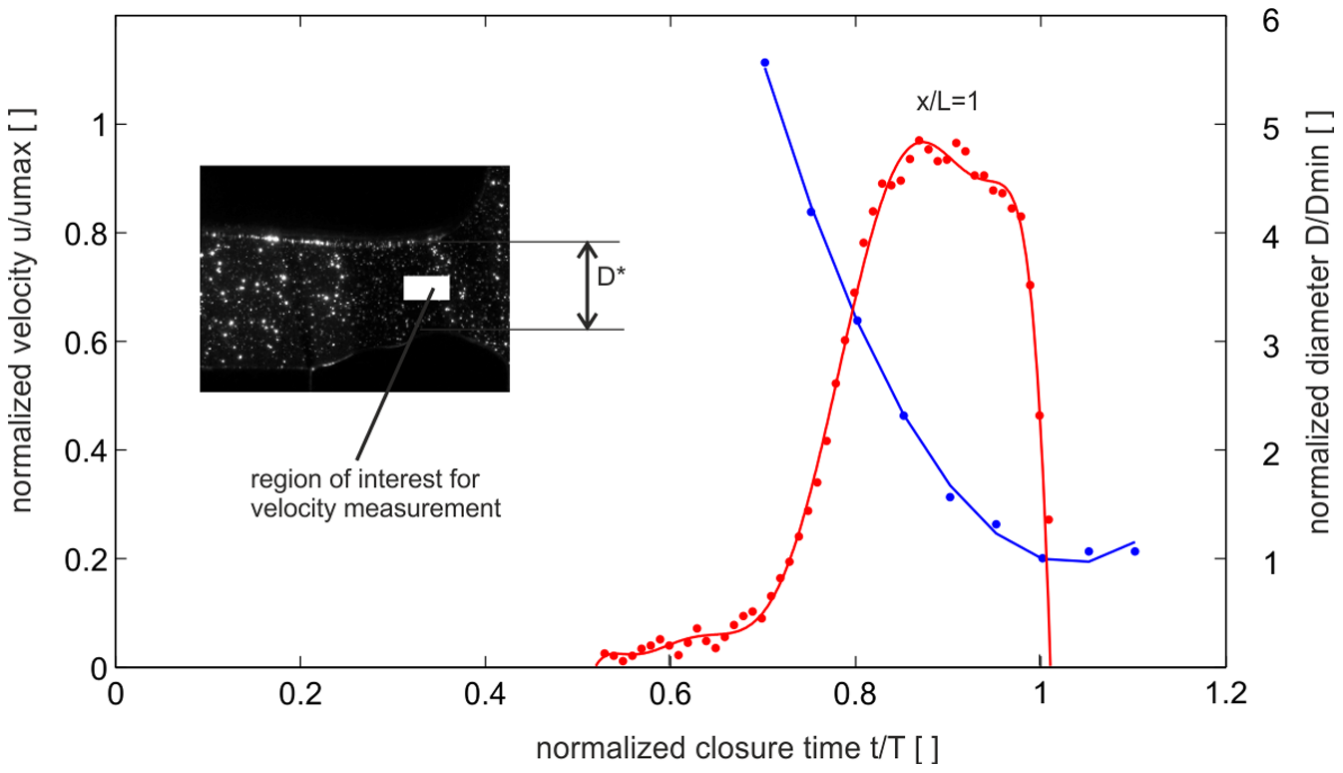
Temporal evolution of the nozzle centre velocity U* (position *x/D* = 1, *y/D* = 0, *U*_max_≅1 m/s) and the nozzle diameter D* (*D*_min_≅1 cm) in the claw closure cycle. The blue curve represents a 2^nd^ order polynomial fit to the nozzle diameter measurements (f(x) = p1*x^2 + p2*x + p3, Coefficients: p1 = 0.4153, p2 = −0.865, p3 = 0.4606).

During closure of the claw, fluid in the gap between plunger and socket is redirected towards the orifice formed by the upper edge of the plunger and the lower floor of the socket. A specific outcome of the geometrical reconstruction is that the formation of the cavity below the plunger during the closure process coincides with the formation of a nozzle-type orifice at the open exit. The orifice has a minimum cross-section where the jet has to pass through before it is ejected into the ambient fluid. The late stage process of the claw closure and the nozzle axis velocity are shown in figure 8, by means the profile of the decreasing exit diameter D* and the velocity evolution at the centre of the nozzle. The curve shows, the nozzle diameter follows a parabolic fit until the final stage, i.e. where the cavity is nearly closed and where the minimum diameter is reached (t* = 1). A small rebound of the claw can be seen, too.

The velocity in the centre of the nozzle increases in a rapid manner in the range of t* = 0.6–0.8, reaches an approximately constant value at t*≈0.9 and finally drops off abruptly from Δt*≈0.04 to zero; at t* = 1.01 it reaches even negative values which are not shown here. Note, that the flow acceleration phase is much longer in comparison to the deceleration phase at the end, where the fluid column in the cavity is abruptly stopped. If at the point of closure a constant deceleration of the fluid column (*dU/dt* = *U/Δt*) is assumed along the length of the cavity *L* within the time-span of Δt*≈0.04 the pressure drop within the cavity can be estimated from Bernoulli's equation as:
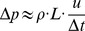
(6)As a consequence of the tension wave, flow reversal near the orifice is observed and negative flow back into the cavity is induced after closure.

**Figure 9:**
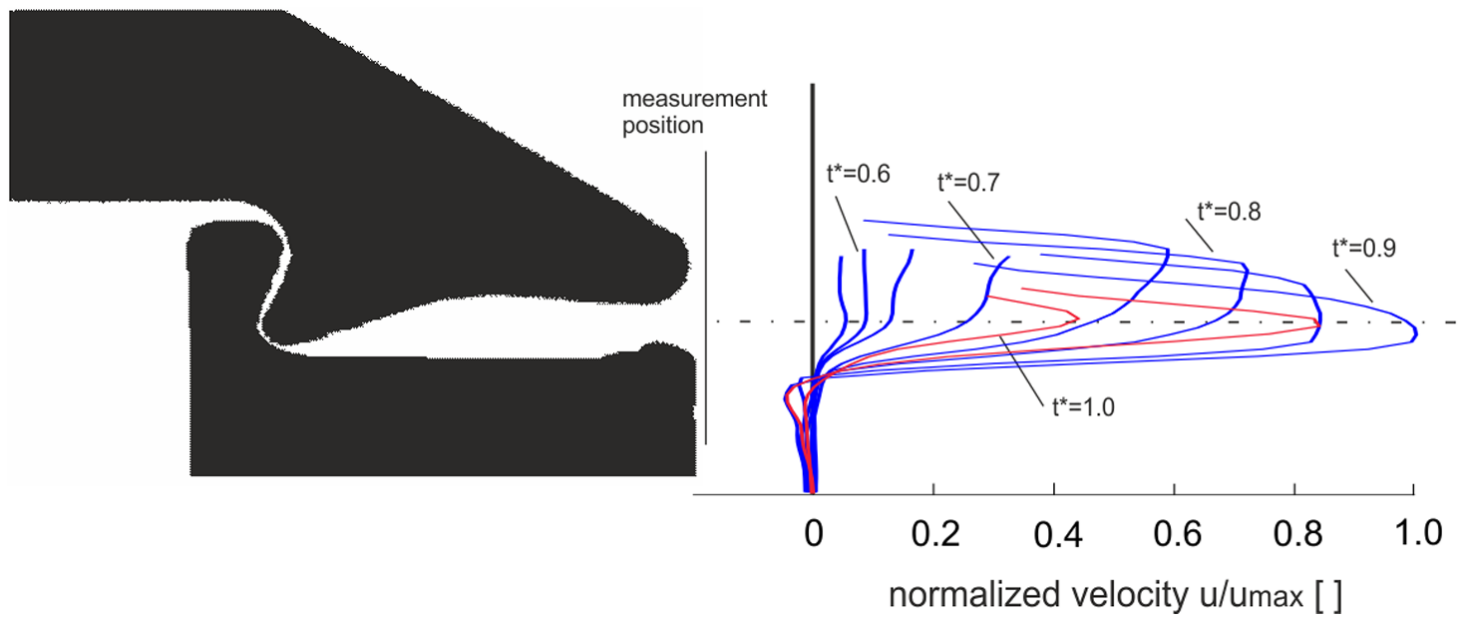
Temporal evolution of the axial velocity profile at the exit of the channel. Blue: acceleration phase, red: deceleration phase.

The velocity profiles at the exit of the channel are shown in figure 9. The blue curves give the profiles at selected points in time with increasing centre velocity, while the red curves show the phase where flow is already in the deceleration phase. Again, peak velocity is reached at t* = 0.9, prior to the end of the closure phase. The velocity profiles indicate a continuous decrease of the jet core diameter during the closure.

**Figure 10:**
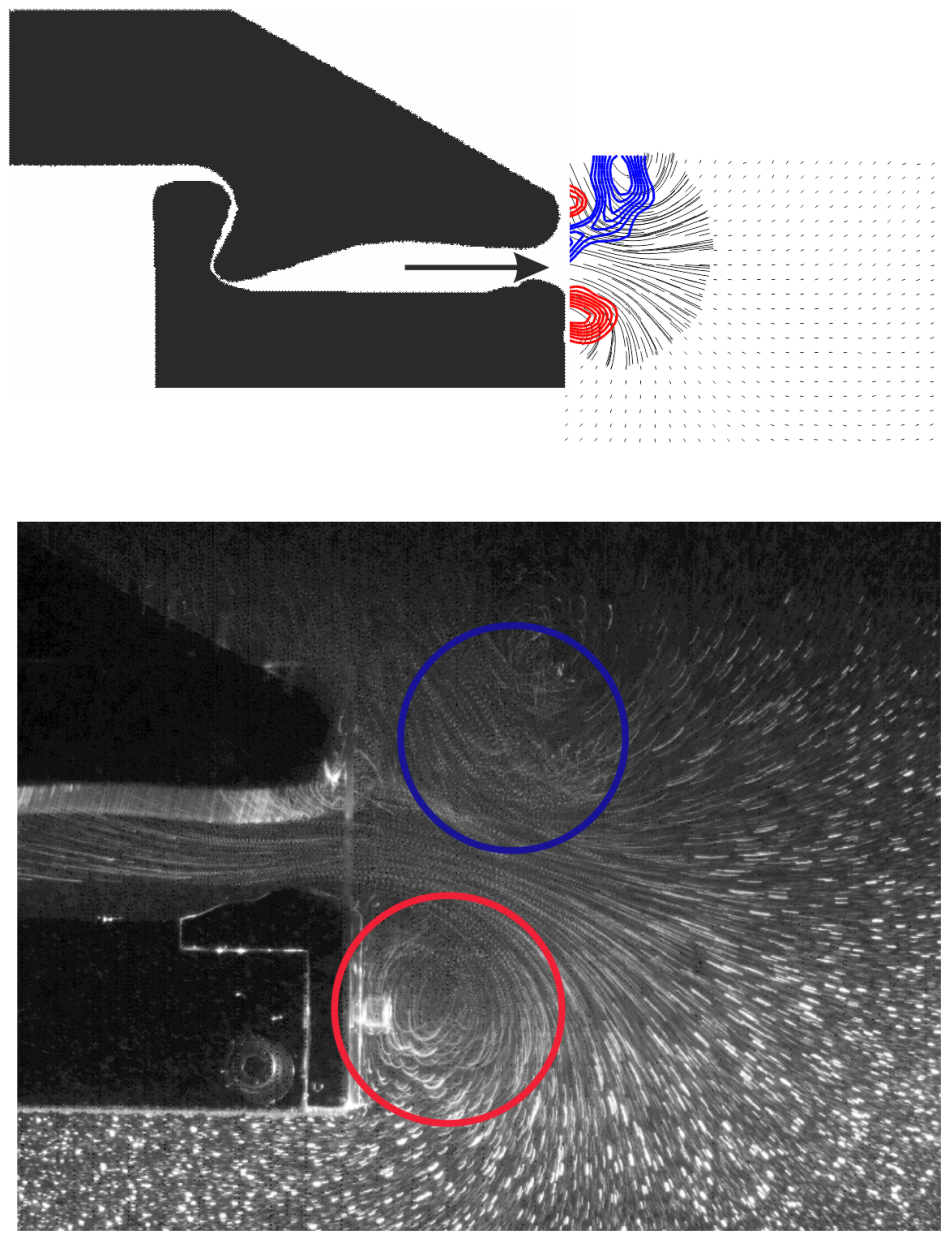
Comparison of the structure of the flow at the exit of the nozzle at t* = 0.95 by means of PIV results (top) and streakline visualization (bottom). PIV results: blue, contours of positive vorticity, red: contours of negative vorticity. Flow visualization: locations of concentrated vorticity regions indicating the structure and position of the vortex ring.

Because the shear layer leaving the cavity is not thin compared to the length scale of axial variations in the nozzle there is a radial pressure gradient which is responsible for the spiral roll-up of the shear layer at the exit of the orifice. This can be seen in figure 10 where the visualization indicates the formation of a vortex on both sides, which are the centre cuts through a ring-like vortex structure. Measurements in other planes (not shown here) were taken to justify this statement of a ring-like structure. A representative plot of the velocity field distribution at the nozzle exit is given in figure 10. Flow and jet penetration is demonstrated by sectional streamlines.

The vortex ring formation is parameterized by the dimensionless vortex formation time *T* and the circulation Γ as a rate of the vorticity, according to the method described by Gharib et al. [6]. The dimensionless time scale that properly accounts for the temporal changes in the exit diameter T* is obtained by integration over the duration of fluid ejection.
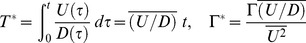
(7)


They investigated the formation of leading vortex rings generated at the orifice of a tube with a piston inside which displaces the fluid in a stroke. When varying the ratio between the piston stroke amplitude (*L*) and the nozzle diameter (*D*) they observed two main types of flows. The first produces a single vortex ring generated by small piston stroke amplitudes. The second produces a combination of a leading vortex ring and trailing vortex rings for higher stroke amplitudes. A transition between both flow regimes appeared at a ratio of *L*/*D* = 4. If this ratio exceeds the critical value of 4 no further circulation is feed into the leading vortex ring. The stroke ratio is also referred to as the formation number ΔT*. This is understood as the time when the leading vortex ring has pinched-off from the trail relative to the characteristic time-scale of the flow 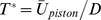 with the bar indicating the time-average value. For flows with time-varying nozzle shape the proper time-scale is given in eq. 7.

**Figure 11:**
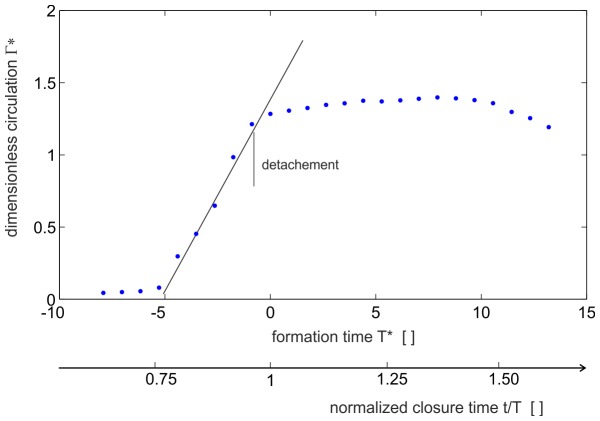
Evaluation process of the vortex ring to compare the formation time and the normalized closure time. The formation number at the complete detachment of the vortex ring from the orifice is ΔT*≈4. No further vorticity is fed into the vortex ring and detachment is observed prior to full closure.

Consistent with the discussion in Dabiri & Gharib [7], the circulation is properly normalized using the terms 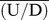 and 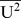, with the bars indicating the time-average of the values. Figure 11 shows the dimensionless circulation versus the formation time. The formation number at the complete detachment of the vortex ring from the orifice is about ΔT*≈4. No further vorticity is fed into the vortex ring and detachment is observed prior to full closure. Within the tail of the vortex ring, some vorticity is seen from the shear-layers at the outer surface of the plunger, which is shed into the wake of the vortex ring. However, this vorticity is much smaller and not accumulated in an orderly manner. As a consequence of the downward motion of the plunger the momentum given to the fluid around the vortex ring also has a vertical component. Thus the travel path of the vortex ring is not along the x-axis but about 30° tilted horizontally in a downward direction. The local pressure minima formed in the core of the vortex near the wall is estimated by the Lamb-vortex [8] as follows:
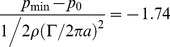
(8)


**Figure 12:**
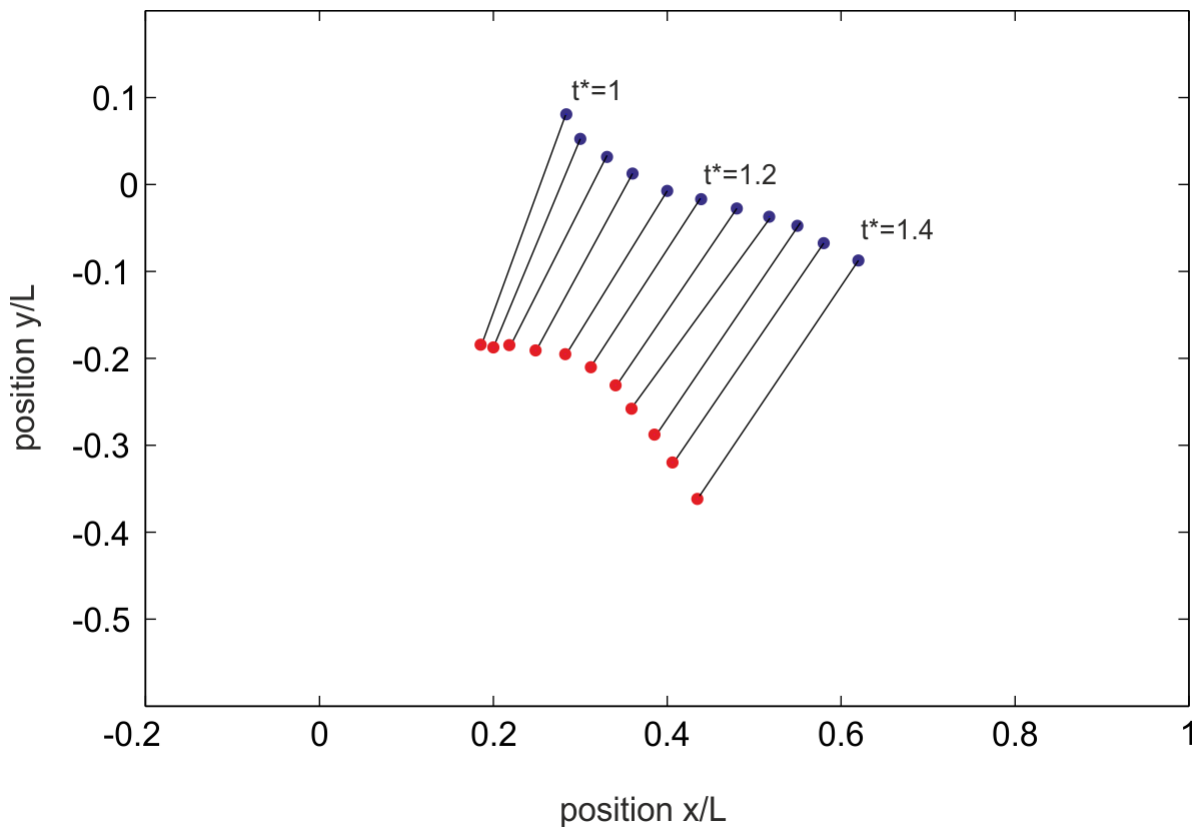
Vortex core positions indicating the traveling and diameter of the vortex ring in the history after the detachment process. Detachment of the vortex ring at t* = 0.96. Thereafter it is first accelerated and shrinks until its minimum is reached at t* = 1.16. During the later stages it approaches a nearly constant traveling speed of about 0.3 *U*.

The vortex strength (circulation) at the time of the pinch-off was approximately Γ≅128 cm^2^/s and the radius of the vortex core was measured at about 4 mm. Both values were taken from the PIV measurements. Thus, the pressure drop in the core was around the order of 200 Pa. This estimated value stands for a vortex ring at zero convection. However, as described above, the detachment of the vortex ring from the orifice was accelerated. In figure 12 the positions of the vortex cores centres are drawn over the detachment process. When the vortex ring detaches from the orifice at normalised closing time t* = 0.96, it is first accelerated and shrinks until its minimum is reached at t* = 1.16. It then approaches a nearly constant traveling speed of about 0.3 *U*. The acceleration of the vortex ring is accompanied by an additional pressure drop, which is proportional to the accelerated fluid mass of the vortex ring including the effect of added mass surrounding the vortex ring (a good approximation is that 50% of the vortex volume is additional accelerated fluid mass). If a constant acceleration (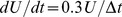) of the vortex ring with a radius *R* = 2 cm is assumed within the time-span of 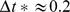, the additional pressure drop according to eq. 6 results to 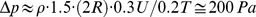. Hence, we can assume a maximum pressure drop in the core of the accelerated vortex ring at about 400 Pa relative to the ambient which is in the order of the dynamic pressure of the jet at maximum velocity within the 70∶1 enlarged model.

### Discussion

An important outcome of the geometrical reconstruction process of the channel formed between the plunger and the socket is that a channel-like cavity is formed with a nozzle-type contour at the orifice. The nozzle at the end of the flow channel accelerates the flow before leaving the orifice with a rounded diffusor-type shape. The curved contours of the outwards-facing walls of the orifice impose a radial pressure gradient where the shear layers roll-up in spirals instead of separating from the wall and leaving the orifice in a straight path. The experiments with the enlarged model clearly demonstrate the formation of an attached vortex ring like structure that grows in time during the flow acceleration period which is the last 20° of plunger motion before the plunger stops in the socket. Maximum velocities in the nozzle reach 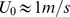
which translates in original scale to a peak velocity of about 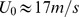.

The continuous decrease in the nozzle exit diameter during the vortex formation counterbalances the deceleration of the plunger such that the shear layers feed the vortex with a near constant circulation flux. Shortly before the end of closure, the vortex ring detaches from the wall at a formation number of 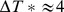
and at maximum strength. The characteristic time-scale used to define the dimensionless formation number is given in eq. (7) and represents the time-average of the ratio of velocity to orifice diameter. This coincides with the deceleration of the fluid in the cavity in the late stage of closure and formation of flow reversal near the orifice. Discussion of the results in light of the conclusions made by Dabiri & Gharib [7] for a vortex ring in a tube with variable exit diameter suggests that the claw might work at the maximum efficiency of the flow ejection process. First, formation number of the vortex ring of 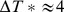
in the experiment herein is in agreement with optimal vortex formation number for circular vortex rings at 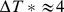, as observed by Gharib et al. [6]. This means that maximum vortex strength is reached and the ring is not able to entrain more vorticity from the jet trail without losing its consistency. Surprisingly, Dabiri [9] found similar optimal vortex formation numbers for a lot of other devices where vortex ring-like structures were generated and even in nature. Second, the claw is generating the vortex ring while the nozzle diameter is decreasing. Closure of the cavity is approximately consistent with the pinch-off of the vortex ring. Thus, with a minimum of ejected volume of fluid the maximum of vortex strength is achieved. This is what Dabiri & Gharib [7] understand as maximum efficiency of vortex formation. Therefore, optimal vortex formation seems to be achieved at the highest efficiency of the flow ejection process while the claw is closing.

**Figure 13:**
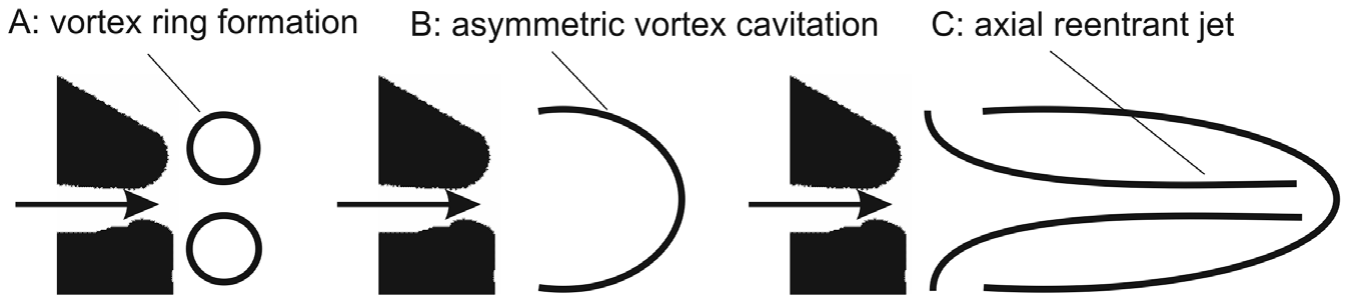
Model to explain the deep penetration depth reached by the initial formation of the leading vortex ring and its subsequent asymmetric cavitation. The formation of an axial reentrant jet that pushes the front of the cylindrical hollow cavity has been documented in detail in [10] for submerged jets.

The analysis of the pressure distribution reveals that the locations of pressure minima in the flow are the vortex core regions. Maximum pressure drop is reached shortly after separation of the vortex ring from the orifice when the vortex ring is accelerated. The amplitude of pressure drop in the vortex core can reach values in the order of the dynamic pressure at maximum flow speed in the nozzle, which is 500 Pa in the model and on the order of 10^5^ Pa in the original organism leading to a cavitation number of Ca<<1. It is therefore suggested, that cavitation inception occurs inside the starting vortex. There might also be a supporting resonance effect by induction of self-excited pressure oscillations of the detaching vortex ring with the fluid in the cavity as described by Arndt [8]. Unfortunately, the 70∶1 enlarged model is not able to reproduce the cavitation process within water at ambient pressure conditions. This would require repeating the experiments in a near-vacuum chamber which was not feasible. However we can estimate the expected behaviour by comparing the cavitation number to other experiments, where cavitating starting vortex rings have been studied in an isolated environment under well-defined boundary conditions of ambient pressure and travel speed. An important feature of the observed cavitating vortex rings - and for the snapping shrimp the most relevant - is the “asymmetric cavitating vortex” as discovered by Belyakov & Filippov [10]. Their study showed symmetric cavitating vortex ring can only exist if the corresponding cavitation number exceeds a certain critical value, which is estimated to be 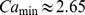. For substantially lower cavitation numbers (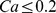), an elongated asymmetric cavitation bubble is generated, with an axial reentrant jet whose length can exceed the initial jet length by several times. This flow structure is called an asymmetric cavitating vortex according to the authors. If we compare the travel speed of the vortex ring of approximately 0.3 *U* in our experiment with the observed disturbance propagation documented in literature for snappers showing cavitation [3], it is obvious that the latter is much faster and reaches much deeper penetration depth in the ambient fluid. The herein estimated pressure drop of the accelerated vortex ring leads to cavitation numbers in the original scale of the snapper that are well in the range of typical cavitation numbers, where asymmetric cavitation of the vortex ring occurs (Ca<0.2). Therefore, it is assumed that vortex cavitation and the built-up of an axial reentrant jet are responsible for the fast disturbance propagation at a speed and penetration length which exceeds those of the starting vortex by several times. An idea of the chronology of the different stages within this jet formation process concluded from the present study is illustrated in figure 13.

What are the behavioural consequences for the animal? Snapping shrimp use water jets to stun or kill prey, and also during ritualized intraspecific agonistic encounters. During fights with conspecifics, they direct jets at each other and assess jet parameters of opponents using hairs on their claws and body. Would vortex ring formation modify (i.e., enhance or reduce) the effectiveness of the jet used as a weapon or fitness signal in inter- and interspecific interactions? Our results suggest that the actual weapon is the strong axial reentrant jet only formed when a sufficient strong leading vortex ring is generated as a precursor that subsequently undergoes asymmetric cavitation after pinch-off. Therefore the optimum formation of the vortex ring during closure of the claw with the given limit of ejected fluid is critical for the effectiveness of the weapon.

The main results can be summarised by the following points:

- The contours of the reconstructed cavity between plunger and socket show, that while closing the gap between plunger and socket a channel-like cavity is formed with a nozzle-type contour at the orifice.
- Flow does not exit the orifice in form of a straight jet but the shear layers roll-up in spirals near the outward facing wall of the orifice and form an attached vortex ring like structure that grows in time.
- Shortly before the end of closure, the vortex ring detaches from the wall at a formation number of 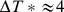 and maximum strength. No further feeding of the ring is observed. Therefore, the detachment is consistent with the pinch-off of the vortex ring from the trail. Thus, maximum angular momentum is achieved.
- The continuous decrease in the nozzle exit diameter during the formation process suggests that the claw might work at the maximum efficiency of the flow ejection process according the conclusion made by Dabiri & Gharib [7] for a vortex ring in a tube with variable exit diameter. Efficiency is understood as the maximum vortex strength achieved by a minimum of fluid volume ejected from the orifice. Hence, the strategy of the snapping shrimp claw is not to generate an initial jet with high axial momentum but to transfer the momentum into maximum strength of the leading vortex ring.
- Maximum pressure drop in the flow is observed in the core of the vortex ring. Within the nozzle the pressure drop can reach values in the order of the dynamic pressure at maximum flow speed. These values are reached shortly after separation of the vortex ring from the orifice when the vortex ring is accelerated. Thus, cavitation numbers as low as Ca≈0.2 can be predicted from the measurements transferred to the original scale in nature. Therefore the pressure drop in the leading vortex is on the order of 10^5^ Pa which is sufficiently high to generate asymmetric cavitation of the vortex ring, compare the results of Belyakov & Filipov [10]. Preliminary test with a downscaled version of the model in 2∶1 scale relative to the snapper's claw show indeed vortex cavitation as predicted. Travel speed and penetration depth of the vortex ring into the ambient fluid is by far lower than the disturbance propagation documented in the literature for snappers. This is a strong hint on the relevance of an axial reentrant jet to reach large penetration depth with the claw. This suggest that the actual weapon is the strong axial reentrant jet that is only formed when a sufficient strong leading vortex ring is generated as a precursor, that subsequently undergoes asymmetric cavitation after pinch-off. Therefore the optimum formation of the vortex ring during closure of the claw with the given limit of ejected fluid is critical for the effectiveness of the weapon.

## Supporting Information

Video S1 **Visualisation of the experiments using long time exposure.** This produces the particle path lines at higher velocities indicating the vortex formation at the orifice of the model claw. The total model closure time is 495 ms and recorded with 300 frames per second.
(MP4)
